# Supplementary material for: Modeling glioblastoma heterogeneity as a dynamic network of cell states
Source: Mol Syst Biol. 2021 Sep 16;17(9):e10105. doi: 10.15252/msb.202010105 (PMC8444284; doi:10.15252/msb.202010105)
Supplement: Supplementary file 5 — Source Data for Figure 3 [file MSB-17-e10105-s001.zip › Figure3A_sourcedata/GSEA_3065/nosigdb_state6.GseaPreranked.1622718443993/RICHARDS_VERHAAK_CANCERCELL_2010_PRONEURAL.html]

Details for gene set RICHARDS\_VERHAAK\_CANCERCELL\_2010\_PRONEURAL[GSEA]

|  || Dataset | state6 |
| Phenotype | NoPhenotypeAvailable |
| Upregulated in class | na\_pos |
| GeneSet | RICHARDS\_VERHAAK\_CANCERCELL\_2010\_PRONEURAL |
| Enrichment Score (ES) | 0.42698914 |
| Normalized Enrichment Score (NES) | 1.8737991 |
| Nominal p-value | 0.0 |
| FDR q-value | 0.0045107505 |
| FWER p-Value | 0.049 |
Table: GSEA Results Summary

  

Fig 1: Enrichment plot: RICHARDS\_VERHAAK\_CANCERCELL\_2010\_PRONEURAL      
 Profile of the Running ES Score & Positions of GeneSet Members on the Rank Ordered List

  

| PROBE | GENE SYMBOL | GENE\_TITLE | RANK IN GENE LIST | RANK METRIC SCORE | RUNNING ES | CORE ENRICHMENT || 1 | SOX4 |  |  | 3 | 0.375 | 0.0511 | Yes |
| 2 | DLL3 |  |  | 49 | 0.246 | 0.0797 | Yes |
| 3 | SOX11 |  |  | 70 | 0.230 | 0.1090 | Yes |
| 4 | OLIG2 |  |  | 126 | 0.201 | 0.1302 | Yes |
| 5 | GADD45G |  |  | 139 | 0.197 | 0.1558 | Yes |
| 6 | TTC3 |  |  | 160 | 0.188 | 0.1792 | Yes |
| 7 | MARCKSL1 |  |  | 223 | 0.169 | 0.1952 | Yes |
| 8 | TMSB15A |  |  | 253 | 0.161 | 0.2140 | Yes |
| 9 | C1QL1 |  |  | 267 | 0.158 | 0.2341 | Yes |
| 10 | CAMSAP2 |  |  | 303 | 0.150 | 0.2507 | Yes |
| 11 | CRMP1 |  |  | 314 | 0.149 | 0.2700 | Yes |
| 12 | PPM1D |  |  | 386 | 0.139 | 0.2809 | Yes |
| 13 | FXYD6 |  |  | 387 | 0.138 | 0.2998 | Yes |
| 14 | TOP2B |  |  | 421 | 0.132 | 0.3142 | Yes |
| 15 | PAFAH1B3 |  |  | 536 | 0.120 | 0.3175 | Yes |
| 16 | SATB1 |  |  | 566 | 0.117 | 0.3301 | Yes |
| 17 | CSNK1E |  |  | 580 | 0.116 | 0.3445 | Yes |
| 18 | KDM1A |  |  | 665 | 0.109 | 0.3497 | Yes |
| 19 | SEZ6L |  |  | 733 | 0.103 | 0.3561 | Yes |
| 20 | GSTA4 |  |  | 745 | 0.102 | 0.3689 | Yes |
| 21 | PAK3 |  |  | 845 | 0.095 | 0.3706 | Yes |
| 22 | SOX2 |  |  | 863 | 0.093 | 0.3814 | Yes |
| 23 | AMOTL2 |  |  | 904 | 0.091 | 0.3892 | Yes |
| 24 | MAP2 |  |  | 917 | 0.090 | 0.4002 | Yes |
| 25 | PFN2 |  |  | 942 | 0.089 | 0.4097 | Yes |
| 26 | TOX3 |  |  | 999 | 0.086 | 0.4150 | Yes |
| 27 | MARCKS |  |  | 1081 | 0.081 | 0.4168 | Yes |
| 28 | KAT7 |  |  | 1090 | 0.081 | 0.4270 | Yes |
| 29 | BEX1 |  |  | 1352 | 0.069 | 0.4064 | No |
| 30 | CKB |  |  | 1359 | 0.069 | 0.4152 | No |
| 31 | GSK3B |  |  | 1382 | 0.068 | 0.4220 | No |
| 32 | ABAT |  |  | 1484 | 0.065 | 0.4192 | No |
| 33 | MYO10 |  |  | 1545 | 0.062 | 0.4208 | No |
| 34 | RUFY3 |  |  | 1615 | 0.060 | 0.4211 | No |
| 35 | STMN1 |  |  | 1649 | 0.058 | 0.4253 | No |
| 36 | SLCO5A1 |  |  | 1875 | 0.051 | 0.4065 | No |
| 37 | CHD7 |  |  | 1953 | 0.049 | 0.4043 | No |
| 38 | ZNF821 |  |  | 1984 | 0.048 | 0.4075 | No |
| 39 | C1orf61 |  |  | 2155 | 0.043 | 0.3939 | No |
| 40 | CSPG5 |  |  | 2321 | 0.038 | 0.3801 | No |
| 41 | MLLT11 |  |  | 2335 | 0.038 | 0.3838 | No |
| 42 | ICK |  |  | 2362 | 0.037 | 0.3859 | No |
| 43 | DCAF7 |  |  | 2575 | 0.032 | 0.3658 | No |
| 44 | FERMT1 |  |  | 2598 | 0.031 | 0.3675 | No |
| 45 | CDC7 |  |  | 2650 | 0.030 | 0.3657 | No |
| 46 | CDC25A |  |  | 2768 | 0.027 | 0.3560 | No |
| 47 | MVB12B |  |  | 2824 | 0.026 | 0.3533 | No |
| 48 | CBX1 |  |  | 2863 | 0.025 | 0.3523 | No |
| 49 | P2RX7 |  |  | 2929 | 0.023 | 0.3480 | No |
| 50 | RBPJ |  |  | 3008 | 0.022 | 0.3421 | No |
| 51 | VEZF1 |  |  | 3022 | 0.022 | 0.3435 | No |
| 52 | MAPT |  |  | 3232 | 0.017 | 0.3218 | No |
| 53 | ATAT1 |  |  | 3237 | 0.017 | 0.3236 | No |
| 54 | CLASP2 |  |  | 3329 | 0.015 | 0.3152 | No |
| 55 | HMGB3 |  |  | 3377 | 0.014 | 0.3117 | No |
| 56 | ARHGEF9 |  |  | 3407 | 0.013 | 0.3101 | No |
| 57 | ZNF184 |  |  | 3634 | 0.008 | 0.2853 | No |
| 58 | MTSS1 |  |  | 3804 | 0.005 | 0.2664 | No |
| 59 | RAP2A |  |  | 3854 | 0.004 | 0.2613 | No |
| 60 | DPYSL4 |  |  | 4057 | -0.000 | 0.2381 | No |
| 61 | PDE10A |  |  | 4180 | -0.003 | 0.2244 | No |
| 62 | TOPBP1 |  |  | 4225 | -0.004 | 0.2199 | No |
| 63 | CASK |  |  | 4430 | -0.007 | 0.1974 | No |
| 64 | CNTN1 |  |  | 4467 | -0.008 | 0.1944 | No |
| 65 | CDKN1B |  |  | 4475 | -0.008 | 0.1947 | No |
| 66 | ERBB3 |  |  | 4525 | -0.009 | 0.1903 | No |
| 67 | MCM10 |  |  | 4638 | -0.011 | 0.1790 | No |
| 68 | ARHGAP33 |  |  | 4663 | -0.012 | 0.1779 | No |
| 69 | YPEL1 |  |  | 4712 | -0.013 | 0.1741 | No |
| 70 | FBXO21 |  |  | 4718 | -0.013 | 0.1753 | No |
| 71 | ZEB2 |  |  | 4735 | -0.013 | 0.1752 | No |
| 72 | WASF1 |  |  | 4764 | -0.014 | 0.1739 | No |
| 73 | DPF1 |  |  | 4782 | -0.014 | 0.1739 | No |
| 74 | GNG4 |  |  | 4886 | -0.016 | 0.1642 | No |
| 75 | MMP15 |  |  | 4887 | -0.016 | 0.1665 | No |
| 76 | BCL7A |  |  | 4912 | -0.017 | 0.1660 | No |
| 77 | RALGPS2 |  |  | 4918 | -0.017 | 0.1677 | No |
| 78 | FHOD3 |  |  | 4940 | -0.017 | 0.1677 | No |
| 79 | EPB41 |  |  | 5038 | -0.019 | 0.1591 | No |
| 80 | ACTR1A |  |  | 5269 | -0.024 | 0.1359 | No |
| 81 | REEP1 |  |  | 5289 | -0.024 | 0.1370 | No |
| 82 | HDAC2 |  |  | 5329 | -0.025 | 0.1359 | No |
| 83 | RNFT2 |  |  | 5448 | -0.027 | 0.1261 | No |
| 84 | DBN1 |  |  | 5587 | -0.030 | 0.1144 | No |
| 85 | BCOR |  |  | 5734 | -0.033 | 0.1021 | No |
| 86 | PODXL2 |  |  | 5938 | -0.037 | 0.0838 | No |
| 87 | PHLPP1 |  |  | 6292 | -0.044 | 0.0493 | No |
| 88 | NLGN3 |  |  | 6305 | -0.045 | 0.0540 | No |
| 89 | NCAM1 |  |  | 6342 | -0.046 | 0.0561 | No |
| 90 | TMCC1 |  |  | 6364 | -0.046 | 0.0600 | No |
| 91 | SEC61A2 |  |  | 6731 | -0.057 | 0.0257 | No |
| 92 | ZNF248 |  |  | 6759 | -0.058 | 0.0305 | No |
| 93 | DPP6 |  |  | 6845 | -0.060 | 0.0290 | No |
| 94 | E2F3 |  |  | 6846 | -0.060 | 0.0372 | No |
| 95 | BCAN |  |  | 6866 | -0.061 | 0.0434 | No |
| 96 | RAD21 |  |  | 6984 | -0.064 | 0.0387 | No |
| 97 | GRIA2 |  |  | 7312 | -0.075 | 0.0114 | No |
| 98 | LRP6 |  |  | 7424 | -0.078 | 0.0093 | No |
| 99 | DNM3 |  |  | 7530 | -0.083 | 0.0086 | No |
| 100 | TSPAN3 |  |  | 7687 | -0.090 | 0.0031 | No |
| 101 | GPM6A |  |  | 7875 | -0.100 | -0.0047 | No |
| 102 | ATAD5 |  |  | 7944 | -0.104 | 0.0016 | No |
| 103 | MMP16 |  |  | 7971 | -0.105 | 0.0131 | No |
| 104 | ADGRL3 |  |  | 8086 | -0.113 | 0.0154 | No |
| 105 | SRGAP3 |  |  | 8135 | -0.117 | 0.0259 | No |
| 106 | ALCAM |  |  | 8323 | -0.135 | 0.0228 | No |
| 107 | TTYH1 |  |  | 8683 | -0.230 | 0.0130 | No |
Table: GSEA details [plain text format]

  

Fig 2: RICHARDS\_VERHAAK\_CANCERCELL\_2010\_PRONEURAL: Random ES distribution      
 Gene set null distribution of ES for **RICHARDS\_VERHAAK\_CANCERCELL\_2010\_PRONEURAL**
